# Survey Language Preference as a Predictor of Meeting Fruit and Vegetable Objectives Among Hispanic Adults in the United States, Behavioral Risk Factor Surveillance System, 2009

**Published:** 2011-10-15

**Authors:** Kirsten A. Grimm, Heidi Michels Blanck

**Affiliations:** Centers for Disease Control and Prevention; Centers for Disease Control and Prevention, Atlanta, Georgia

## Abstract

**Introduction:**

Although Hispanics are a rapidly growing ethnic minority in the United States, the effect of acculturation on the proportion of Hispanics who meet national objectives for fruit and vegetable consumption has not been fully investigated. Our objective was to determine the extent to which ethnicity and acculturation (indicated by survey language preference) are associated with fruit and vegetable consumption among Hispanics in the United States.

**Methods:**

Fruit and vegetable consumption among adult respondents to the 2009 Behavioral Risk Factor Surveillance System was determined from data collected from the 31 states and 2 territories that offered the fruit and vegetable screener in Spanish and English (n = 287,997). Logistic regression analyses were used to determine whether ethnicity (Hispanic vs non-Hispanic white) and survey language preference (English vs Spanish) were related to meeting objectives of consuming fruit 2 or more times per day and vegetables 3 or more times per day.

**Results:**

More Hispanics (37.6%) than non-Hispanic whites (32.0%) and more Spanish-speaking Hispanics (41.0%) than English-speaking Hispanics (34.7%) ate fruit 2 or more times per day. Conversely, more non-Hispanic whites (28.5%) than Hispanics (18.9%) and more English-speaking Hispanics (21.8%) than Spanish-speaking Hispanics (15.8%) ate vegetables 3 or more times per day. All associations remained significant after controlling for covariates.

**Conclusion:**

Our findings have implications regarding how brief screeners can be used to determine possible dietary disparities among the Hispanic population in the United States and to monitor population goals to eliminate racial and ethnic health disparities.

## Introduction

Higher dietary intake of fruits and vegetables is associated with a lower risk for chronic disease and can benefit weight management (1-4). *Healthy People* *2010* objectives were for 75% of the population aged 2 years or older to consume 2 or more daily servings of fruit and 50% of the population aged 2 years or older to consume 3 or more daily servings of vegetables (5). These objectives replaced the earlier *Healthy People 2000* objective of consuming 5 or more servings of fruits and vegetables daily (6).

Hispanics are a rapidly growing ethnic minority in the United States. In 2009, approximately 16% of the population (48.4 million people) was Hispanic (7). Acculturation, the process through which immigrants assume the dominant characteristics of the society to which they immigrate, may be gauged through either multidimensional acculturation scales or a single dimension (eg, primary language spoken, years of residence). Acculturation has been associated with overall decline in dietary quality and, particularly among Hispanics, decreased health status with increased acculturation (8-10). Increased acculturation is associated with decreased intake of fruits and vegetables, as determined by food frequency screeners (11,12), 24-hour dietary recall (13,14), and serum carotenoid levels (14). Few studies have assessed intake of fruits and vegetables separately.

Monitoring health behaviors, including dietary intake, by ethnicity may elucidate health disparities and help determine progress toward national goals to eliminate racial and ethnic health disparities. Monitoring residents' fruit and vegetable intake via the state-based Behavioral Risk Factor Surveillance System (BRFSS) is a way to assess progress toward *Healthy People* objectives (5). Previous analyses of BRFSS among Hispanics by survey language preference, used as a measure of acculturation, have assessed health awareness and behaviors and access to health care (15-18), but, to our knowledge, none have used BRFSS survey language preference to assess the effect of acculturation on fruit and vegetable intake (15-18).

We sought to compare the proportion of Hispanic versus non-Hispanic whites who met objectives for fruit and vegetable consumption, assess the proportion of Hispanic adults who met objectives for fruit and vegetable consumption by survey language preference (Spanish vs English), and determine the extent to which ethnicity and acculturation, as indicated by survey language preference, were associated with meeting these objectives.

## Methods

**Box. Six-Item Screener to Assess Fruit and Vegetable Consumption, Behavioral Risk Factor Surveillance System, 2009**

**Table:** 

These next questions are about the foods you usually eat or drink. Please tell me how often you eat or drink each one, for example, twice a week, three times a month, and so forth. Remember, I am only interested in the foods *you* eat. Include all foods you eat, both at home and away from home.
1. How often do you drink fruit juices such as orange, grapefruit, or tomato?
2. Not counting juice, how often do you eat fruit?
3. How often do you eat green salad?
4. How often do you eat potatoes not including French fries, fried potatoes, or potato chips?
5. How often do you eat carrots?
6. Not counting carrots, potatoes, or salad, how many servings of vegetables do you usually eat?

BRFSS is a random-digit–dialed telephone survey conducted by the Centers for Disease Control and Prevention (CDC) that collects health risk data from all 50 states, the District of Columbia, Guam, Puerto Rico, and the Virgin Islands. The target population is noninstitutionalized people aged 18 years or older with access to a landline telephone. Cross-sectional data are collected on behaviors, health care access, and chronic disease status (www.cdc.gov/brfss). The BRFSS survey questionnaire contains a 6-item screener to assess usual frequency of consumption of fruits and vegetables (Box); participants are not given a definition of a serving size. We calculated total daily frequency of fruit consumption from responses to questions 1 and 2 and total daily frequency of vegetable consumption from responses to questions 3 through 6.

An optional Spanish language version of the BRFSS questionnaire is available for all states, and its use has increased over time. In 2009, 31 states (Alaska, Arizona, Arkansas, California, Colorado, Connecticut, Florida, Georgia, Hawaii, Idaho, Illinois, Indiana, Iowa, Kansas, Maryland, Massachusetts, Montana, Nebraska, Nevada, New Jersey, New Mexico, New York, North Carolina, Oklahoma, Oregon, Rhode Island, Texas, Utah, Virginia, Washington, and Wyoming) and 2 territories (Puerto Rico and the Virgin Islands) used the Spanish language module (19). We limited our sample to people who lived in states that administered the Spanish language questionnaire (N = 287,997). We excluded respondents if they did not specify survey language (n = 2,197), were missing more than 1 screener question (n = 17,467), or had unlikely daily values for fruits and vegetables (ie, ≥25) (n = 120). Following exclusions, 236,231 respondents had a self-reported ethnic status of non-Hispanic white (n = 211,045) or Hispanic (n = 25,186). A smaller subsample for acculturation analysis comprised Hispanics who identified completing the questionnaire in either Spanish (n = 11,141) or English (n = 11,848). Multivariable logistic regression analyses were limited to respondents with complete information on all covariates.

Descriptive statistics and logistic regression analyses were performed using SAS 9.1 and SAS-Callable SUDAAN (RTI International, Research Triangle Park, North Carolina) to account for the complex survey design. Separate multivariable logistic regression models were used to assess the proportion of adults consuming daily: ≥2 fruits (objective 19-5) and ≥3 vegetables (objective 19-6). These outcome measures were derived from the *Healthy People 2010* objectives. We conducted logistic regression analyses to obtain crude odds ratios (ORs) and to adjust for multiple covariates including sex, age, education, annual household income, employment status, marital status, household number, region, health care access, and personal physician. To determine whether the proportion who met the objectives differed by sociodemographic factors, we obtained prevalence estimates and 95% confidence intervals (CIs). Because of nonnormal distribution, we also provide the median and interquartile range for intake of fruits, vegetables, and each of the 6 screener items.

## Results

Variation in sample characteristics was found by ethnicity and survey language preference (Table 1). Compared with Hispanics, a lower proportion of non-Hispanic whites were aged 18 to 34 years, and a higher proportion were more educated, had an annual household income at or above $50,000, and lived in households with only 1 or 2 members. More Hispanics lived in the West and territories than did non-Hispanic whites.

The distribution of sex did not vary widely by language preference among Hispanics. A higher proportion of Hispanic respondents with an English survey language preference compared with Spanish language preference were aged 18 to 34 years, were more educated, had an annual household income at or above $25,000, lived in households with 3 or fewer members, and resided in the South. Conversely, a greater proportion of Spanish-speaking Hispanics were aged 35 to 54 years and lived in the territories compared with English-speaking Hispanics (18.9% vs <1.0%) (Table 1).

**Figure 1.**
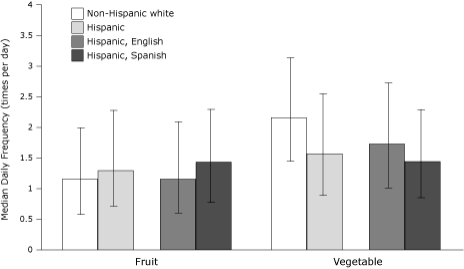
Median daily frequency (times per day) of fruit and vegetable consumption among participants who completed the fruit and vegetable screener of the 2009 Behavioral Risk Factor Surveillance System (bars denote interquartile ranges).

Overall, few non-Hispanic white and Hispanic respondents met the *Healthy People 2010* objectives for fruit and vegetable consumption (5). Compared with non-Hispanic whites (32.0%), more Hispanics (37.6%) ate fruit 2 or more times per day (Table 2). Conversely, more non-Hispanic whites (28.5%) than Hispanics (18.9%) ate vegetables 3 or more times per day. In the unadjusted model, Hispanics were more likely than non-Hispanic whites to eat fruit 2 or more times per day, and non-Hispanic whites were more likely than Hispanics to eat vegetables 3 or more times per day. These overall differences remained significant after adjustment for multiple covariates (Table 3). Findings were similar with regard to median daily fruit and vegetable consumption (Figure 1).

**Table:** 

**Ethnicity/Survey Language Preference**	**Median**	**Interquartile Range**	
**Non-Hispanic white**			
Fruit ≥ 2	1.14	0.58	1.99
Veg ≥ 3	2.16	1.45	3.14
**Hispanic**			
Fruit ≥ 2	1.28	0.71	2.28
Veg ≥ 3	1.57	0.89	2.56
**English survey language preference**			
Fruit ≥ 2	1.14	0.6	2.1
Veg ≥ 3	1.72	1	2.72
**Spanish survey language preference**			
Fruit ≥ 2	1.42	0.78	2.29
Veg ≥ 3	1.43	0.85	2.29

Slightly more Spanish-speaking Hispanics (41.0%) than English-speaking Hispanics (34.7%) met the fruit objective (Table 2). Conversely, more English-speaking Hispanics (21.8%) met the vegetable objective than did Spanish-speaking Hispanics (15.8%). In the unadjusted model, Spanish-speaking Hispanics were more likely to meet the fruit objective than were English-speaking Hispanics, and English-speaking Hispanics were more likely to meet the vegetable objective than were Spanish-speaking Hispanics. These overall differences in fruit and vegetable consumption by acculturation remained significant after adjusting for covariates (Table 3).

For comparison to the historically used 5-A-Day measure from *Healthy People 2000* (6), which does not distinguish between consumption of fruits or vegetables, we found that the consumption of 5 or more fruits or vegetables per day was higher among non-Hispanic whites (25.1%) than Hispanics (22.3%) (OR = 0.86; 95% CI, 0.81-0.91). However, the prevalence among Hispanics of consuming 5 or more fruits or vegetables per day was higher among English-speaking Hispanics (23.9%) than Spanish-speaking Hispanics (20.9%) (OR = 0.84; 95% CI, 0.75-0.95) (data not shown).

**Figure 2.**
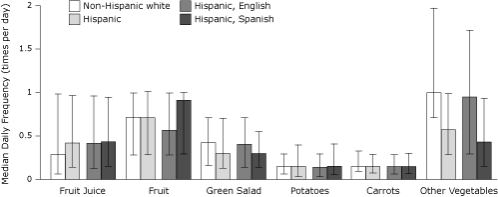
Median daily frequency (times per day) of consumption of specific fruit and vegetable items among participants who completed the fruit and vegetable screener of the 2009 Behavioral Risk Factor Surveillance System (bars denote interquartile ranges).

Consumption of whole fruit, nonfried potatoes, and carrots was similar among Hispanics and non-Hispanic whites (Figure 2). However, consumption of fruit juice was higher among Hispanics than non-Hispanic whites, and consumption of green salad and other vegetables was lower among Hispanics than non-Hispanic whites. Consumption of fruit juice, carrots, and nonfried potatoes was similar among Spanish-speaking and English-speaking Hispanics. However, compared with English-speaking Hispanics, Spanish-speaking Hispanics reported a higher consumption of whole fruit and a lower consumption of green salad and other vegetables (Figure 2).

**Table:** 

**Ethnicity/Survey Language Preference**	**Median**	**Interquartile Range**	
**Non-Hispanic white**			
Fruit Juice	0.28	0.06	0.98
Fruit	0.71	0.28	0.99
Green Salad	0.42	0.16	0.71
Potatoes	0.14	0.06	0.29
Carrots	0.14	0.09	0.32
Otder	0.99	0.71	1.96
**Hispanic**			
Fruit Juice	0.41	0.13	0.96
Fruit	0.7	0.28	1
Green Salad	0.29	0.13	0.7
Potatoes	0.14	0.03	0.4
Carrots	0.14	0.07	0.28
Other	0.56	0.28	0.99
**English survey language preference**			
Fruit Juice	0.41	0.13	0.96
Fruit	0.56	0.28	0.99
Green Salad	0.4	0.14	0.7
Potatoes	0.13	0.03	0.29
Carrots	0.14	0.06	0.28
Other	0.94	0.29	1.71
**Spanish survey language preference**			
Fruit Juice	0.42	0.14	0.94
Fruit	0.9	0.29	0.99
Green Salad	0.29	0.13	0.55
Potatoes	0.14	0.05	0.41
Carrots	0.14	0.06	0.29
Other	0.42	0.14	0.93

## Discussion

In keeping with findings from previous studies of US adults, we found that both non-Hispanic whites and Hispanics failed to meet *Healthy People 2010* targets for consumption of both fruits and vegetables. Although more Hispanics than non-Hispanic whites met the fruit objective (+5.6%), more non-Hispanic whites than Hispanics met the vegetable objective (−9.6%).

Our results indicated that more Spanish-speaking Hispanics met the fruit objective than did English-speaking Hispanics (+6.3%). The acculturation effect on fruit consumption, as measured by survey language preference, was strengthened following adjustment for sociodemographic variables in regression models. The findings for lower fruit consumption among English-speaking Hispanics are similar to those from the National Cancer Institute 7-item fruit and vegetable screener, which indicated that greater language acculturation among Mexican-American women was associated with significantly decreased fruit consumption (11).

Conversely, fewer Spanish-speaking Hispanics met the vegetable objective than did English-speaking Hispanics (−6.0%). The effect of acculturation, as measured by survey language preference, on vegetable consumption remained significant in regression models after accounting for sociodemographic variables. Findings for fruit (34.7%) and vegetable (21.8%) consumption among English-speaking Hispanics were intermediate to intake among non-Hispanic whites and Spanish-speaking Hispanics, which is in keeping with the expectation that increased acculturation will yield dietary patterns more aligned with those of the population majority.

A more detailed comparison of screener items indicated some differential consumption by ethnicity and survey language preference, particularly regarding vegetable intake. Our findings may reflect true differences in vegetable consumption influenced by potential factors such as access and affordability, or they may reflect the ability of the translated screener to adequately query about vegetables categorized as "green salad" or "other vegetables." Small but significant differences in individual components of intake across Hispanic subgroups were also found in the 2005 California Health Interview Survey (CHIS), which used a short 7-item dietary screener similar to BRFSS (fruit juice, fruit, green salad, cooked-dried beans, fried potatoes, nonfried potatoes, and other vegetables). Specifically, intake of "other white potatoes" was different across subgroups of Hispanic women. Consumption of green salad also varied among women; South American women reported higher intake of green salad than did Central American women (20). Results from CHIS suggest that dietary preferences differ among heterogeneous Hispanic subgroups, an aspect that state-specific analyses of BRFSS data may be able to further explore. Future research may investigate the reasons for the variation in fruit and vegetable intake, particularly since evidence supports decreased differential risk for cancer attributed to fruits compared with vegetables (21).

Furthermore, because of recent overall changing dietary patterns in Mexico and Latin America, the premise that diet quality among Hispanics living in the United States decreases with increased acculturation may no longer hold true. In Mexico, findings from health and nutrition surveys have found temporal changes in dietary intake (22), and results from multiple national nutrition surveys conducted among adult residents in countries in Latin America and the Caribbean indicate that consumption of fruits, vegetables, grains, cereals, and legumes has decreased and that consumption of saturated fat has increased (23).

Assessment of fruit and vegetable intake using BRFSS has strengths as well as some limitations. Strengths of this study include using a representative population-based sample, including respondents from territories, and a large sample size. Although fruits and vegetables are just 1 part of a healthy diet, they are foods encouraged by the *Dietary Guidelines for Americans 2010* (24) and are the only continuously monitored nutritional intake items in the state-based BRFSS. Compared with other dietary components that influence diet quality, as measured by the Healthy Eating Index 2005, whole fruit consumption has the highest correlation with overall dietary quality (*r* = 0.45 for whole fruit, *r* = 0.43 for total fruit, *r* = 0.18 for vegetables, *r* = 0.07 for total grains, and *r* = −0.12 for milk) (25).

We were restricted to survey language preference as the only measure by which to assess acculturation. Determining acculturation through assessment scales in population-based surveys such as BRFSS may be difficult due to limitations on the number of survey items to reduce respondent burden and maximize participation. However, a strong correlation was found (*r* = 0.80) between a 1-item language preference question on a telephone survey and a validated acculturation assessment instrument among Latino adults, validating the use of survey language preference as a proxy for acculturation in this population (26).

Although CDC provides a Spanish translation of the BRFSS survey, there may be differences in how individual states and territories translate the screener for use in their jurisdictions, depending on population characteristics of the majority of Spanish-speaking residents in that state. Adding a question about country of origin on the BRFSS may be a future consideration to address heterogeneity issues among respondents in this population. A preliminary report from the National Health Interview Survey for 2007 found that Hispanics or Latinos had a higher percentage (18.0%-19.3%) of households that were wireless-only (eg, residents used only cellular telephones) compared with non-Hispanic whites (11.3%-12.9%) (16,27). To our knowledge, assessment of sociodemographic characteristics by race/ethnicity for cellular telephone users has not been published, and we are unsure how this may affect our estimates.

Finally, estimates calculated on the basis of abbreviated food frequency questionnaires, such as the BRFSS fruit and vegetable screener, are generally lower than those from studies that use other methods such as the National Health and Nutrition Examination Survey 24-hour recall, which limits comparison across surveys (28-30). However, for the purpose of surveillance, the module has moderate validity when compared with other dietary assessment methods (29). The exclusion of fried potatoes, such as French fries, in the BRFSS fruit and vegetable screener also contributes to lower estimates of overall intake.

Few assessments of surveillance data have determined differences in dietary intake by acculturation as measured by survey language preference. Although a causal relationship cannot definitively be established between acculturation and fruit and vegetable intake, our findings have implications regarding acculturation among the Hispanic population in the United States. Our analysis demonstrates how brief screeners can be used to determine possible disparities among minority groups and to monitor population goals to eliminate racial and ethnic health disparities.

## Figures and Tables

**Table 1.** Respondent Characteristics, by Ethnicity and Survey Language Preference, for the 31 States and 2 Territories Using the Spanish Language Module, Behavioral Risk Factor Surveillance System, 2009

Characteristic	Ethnicity		Survey Language Preference Among Hispanics	

	Non-Hispanic White (N = 211,045), No. (%)^a^	Hispanic (N = 25,186), No. (%)	English (N = 11,848), No. (%)	Spanish (N = 11,141), No. (%)
**Sex**				
Male	82,369 (48.2)	9,045 (49.8)	4,315 (50.3)	3,914 (49.4)
Female	128,676 (51.9)	16,141 (50.2)	7,533 (49.7)	7,227 (50.6)
**Age, y**				
18-34	20,870 (26.0)	6,055 (43.1)	3,030 (45.8)	2,629 (40.3)
35-54	70,294 (37.9)	10,351 (38.7)	4,871 (36.5)	4,627 (41.2)
55-64	48,891 (16.2)	4,039 (9.7)	1,936 (9.8)	1,668 (9.7)
≥65	70,990 (19.9)	4,741 (8.5)	2,011 (8.0)	2,217 (8.9)
**Education**				
<High school	11,891 (5.4)	7,720 (31.5)	1,789 (13.3)	5,452 (50.8)
High school graduate	58,882 (25.7)	7,111 (27.8)	3,768 (30.2)	2,633 (25.1)
Some college	59,677 (27.7)	5,477 (22.3)	3,407 (31.2)	1,491 (12.9)
College graduate	80,371 (41.2)	4,817 (18.4)	2,862 (25.3)	1,526 (11.2)
**Annual household income, $**				
<25,000	42,651 (17.8)	11,623 (50.4)	3,840 (32.9)	6,883 (69.4)
25,000-49,999	51,150 (23.3)	5,484 (25.8)	3,024 (27.3)	1,906 (24.0)
≥50,000	93,056 (59.0)	4,845 (23.8)	3,803 (39.8)	541 (6.7)
**Employment**				
Salaried	88,864 (49.7)	10,629 (47.0)	5,664 (50.0)	4,065 (44.0)
Self-employed	20,130 (9.2)	1,689 (7.5)	821 (6.85)	696 (8.1)
Unemployed/unable to work	20,883 (10.9)	4,444 (18.1)	2,070 (19.1)	1,982 (17.0)
Homemaker/retired	78,096 (26.0)	7,587 (20.6)	2,798 (14.5)	4,124 (26.9)
Student	2,657 (4.1)	774 (6.9)	464 (9.6)	254 (4.0)
**Number in household**				
1	60,238 (13.2)	4,392 (5.6)	2,180 (6.9)	1,744 (4.1)
2	84,304 (34.7)	6,632 (16.2)	3,365 (19.7)	2,536 (12.4)
3	26,385 (18.5)	4,361 (18.9)	2,144 (21.6)	1,886 (16.1)
4	24,101 (19.5)	4,674 (23.7)	2,131 (23.8)	2,199 (23.8)
5	10,352 (9.0)	2,873 (16.8)	1,186 (14.4)	1,495 (19.4)
≥6	5,665 (5.1)	2,254 (18.8)	842 (13.6)	1,281 (24.2)
**Region**				
Northeast	35,780 (18.6)	3,517 (12.6)	2,039 (17.2)	1,450 (8.1)
Midwest	47,259 (14.9)	1,828 (5.3)	1,211 (7.7)	617 (3.2)
South	48,121 (37.1)	4,623 (29.6)	2,902 (34.9)	1,721 (25.2)
West	79,442 (29.3)	11,044 (43.3)	5,501 (40.2)	3,374 (44.6)
Territories	443 (0.03)	4,174 (9.2)	195 (0.05)	3,979 (18.9)

a Numbers may not total to 100 due to missing data.

**Table 2.** Proportion of Respondents Who Met Objectives for Fruit and Vegetable Consumption, by Ethnicity and Survey Language Preference Subgroup, BRFSS, 2009^a^

**Subgroup**	Consumed ≥2 Fruits/Day, % (95% CI)	Consumed ≥3 Vegetables/Day, % (95% CI)
Non-Hispanic white	32.0 (31.6-32.4)	28.5 (28.2-28.9)
Hispanic	37.6 (36.4-38.8)	18.9 (18.0-19.8)
Hispanic, English language survey preference	34.7 (33.0-36.4)	21.8 (20.5-23.3)
Hispanic, Spanish language survey preference	41.0 (39.3-42.7)	15.8 (14.6-17.0)

Abbreviation: BRFSS, Behavioral Risk Factor Surveillance System; CI, confidence interval.

a Derived from *Healthy People* *2010* objectives: 75% of the population aged ≥2 y should consume ≥2 servings/day of fruit (19-5) and 50% of the population aged ≥2 y should consume ≥3 servings/day of vegetables (19-6) (5). BRFSS assessed frequency of fruit and vegetable consumption (ie, how often) and not servings/day.

**Table 3.** Odds of Meeting Objectives for Fruit and Vegetable Consumption, by Ethnicity and Survey Language Preference Subgroup, BRFSS, 2009^a^

Subgroup	Consumed ≥2 Fruits/Day		Consumed ≥3 Vegetables/Day	

	Model 1,^b^ OR (95% CI)	Model 2,^b^ AOR (95% CI)	Model 1,^b^ OR (95% CI)	Model 2,^b^ AOR (95% CI)
Non-Hispanic white	1 [Reference]			
Hispanic	1.28 (1.21-1.35)	1.50 (1.40-1.60)	0.58 (0.55-0.62)	0.75 (0.70-0.82)
Hispanic, ELSP	1 [Reference]			
Hispanic, SLSP	1.31 (1.18-1.45)	1.42 (1.22-1.65)	0.67 (0.59-0.76)	0.82 (0.69-0.98)

Abbreviations: BRFSS, Behavioral Risk Factor Surveillance System; OR, odds ratio; CI, confidence interval; AOR, adjusted odds ratio; ELSP, English language survey preference; SLSP, Spanish language survey preference.

a Derived from *Healthy People* *2010* objectives: 75% of the population aged ≥2 y should consume ≥2 servings/day of fruit (19-5) and 50% of the population aged ≥2 y should consume ≥3 servings/day of vegetables (19-6) (5). BRFSS assessed frequency of fruit and vegetable consumption (ie, how often) and not servings/day.

b Model 1 is unadjusted; model 2 adjusted for sex, age, education, annual household income, employment status, marital status, household number, region, health care access, and personal physician.
